# Identification of patients with and without minimal hepatic encephalopathy based on gray matter volumetry using a support vector machine learning algorithm

**DOI:** 10.1038/s41598-020-59433-1

**Published:** 2020-02-12

**Authors:** Qiu-Feng Chen, Tian-Xiu Zou, Zhe-Ting Yang, Hua-Jun Chen

**Affiliations:** 1 College of Computer and Information Sciences, Fujian Agriculture and Forestry University, Fuzhou, 350002 China; 2 Department of Radiology, Fujian Medical University Union Hospital, Fuzhou, 350001 China

**Keywords:** Cognitive neuroscience, Computational neuroscience, Learning and memory, Computational biology and bioinformatics, Neuroscience, Neurology

## Abstract

Minimal hepatic encephalopathy (MHE) is characterized by diffuse abnormalities in cerebral structure, such as reduced cortical thickness and altered brain parenchymal volume. This study tested the potential of gray matter (GM) volumetry to differentiate between cirrhotic patients with and without MHE using a support vector machine (SVM) learning method. High-resolution, T1-weighted magnetic resonance images were acquired from 24 cirrhotic patients with MHE and 29 cirrhotic patients without MHE (NHE). Voxel-based morphometry was conducted to evaluate the GM volume (GMV) for each subject. An SVM classifier was employed to explore the ability of the GMV measurement to diagnose MHE, and the leave-one-out cross-validation method was used to assess classification accuracy. The SVM algorithm based on GM volumetry achieved a classification accuracy of 83.02%, with a sensitivity of 83.33% and a specificity of 82.76%. The majority of the most discriminative GMVs were located in the bilateral frontal lobe, bilateral lentiform nucleus, bilateral thalamus, bilateral sensorimotor areas, bilateral visual regions, bilateral temporal lobe, bilateral cerebellum, left inferior parietal lobe, and right precuneus/posterior cingulate gyrus. Our results suggest that SVM analysis based on GM volumetry has the potential to help diagnose MHE in cirrhotic patients.

## Introduction

Cirrhotic patients with liver dysfunction often exhibit alterations in brain structure. Neuronal cell loss was shown to be associated with liver failure and is likely induced by chronic portosystemic shunting and ammonia exposure^1^. Many neuropathological studies have reported that cirrhosis involves a loss of brain parenchyma^2^. Previous findings from *in vivo* computed tomography studies also revealed brain atrophy in alcoholic and non-alcoholic cirrhosis^3,4^. In addition, Zeneroli and colleagues^5^ showed compelling evidence of brain atrophy in cirrhotic patients with hepatic encephalopathy (HE) using magnetic resonance imaging (MRI). Quite recently, reductions in the density of gray matter and white matter in cirrhotic patients without overt HE were reported^6^. From the findings above, we see that the literature has repeatedly documented a decline in brain mass that accompanied advanced liver disease^3,6^. Additionally, it was implied that brain structural impairments may increase susceptibility to various neurotoxic substances derived from hepatic dysfunction-associated metabolic disorders, such as ammonia and manganese^7^. More importantly, these structural alterations were suggested to be associated with abnormal brain electrophysiological activity and poor psychometric performance in cirrhotic patients^8^. Therefore, measurement of structural alterations in the brain may be helpful to assess potential brain dysfunction in cirrhotic patients.

As an early stage of HE, minimal HE (MHE) represents the mildest form of neuropsychological dysfunction related to cirrhosis. The symptoms of MHE include an array of mild neurocognitive impairments, such as psychomotor retardation, memory impairments, attention deficits, and diminished executive function^9^. There is increasing evidence showing that MHE negatively affects quality of life^10^, impairs driving capabilities^11^, predicts the development of overt HE^12^, and heightens the risk of death^13,14^. The clinical manifestations of MHE are too mild to be identified by routine physical and neurological examinations. MHE patients are often misdiagnosed or left untreated because their subtle neurocognitive impairments require specific neuropsychological and neurophysiological tests to be detected^15^.

Notably, the structural abnormalities occurring in the gray matter (GM) are considered to contribute to the neuropsychological dysfunction in MHE and have been associated with the progression of HE^6,16,17^. Several studies even proposed that regional GM morphometry (such as regional volume and cortical thickness measurements) could help to predict the existence of MHE^18,19^. Given these findings, we used a support vector machine (SVM) learning method to test the extent to which GM volumetry can distinguish between cirrhotic patients with and without MHE. Additionally, this study aimed to identify the specific GM regions that contributed the most to differentiating between the two patient groups.

## Subjects and Methods

### Subjects

This study was approved by the Research Ethics Committee of Fujian Medical University Union Hospital and was conducted in accordance with the Declaration of Helsinki. Written informed consent was obtained from all the study subjects: cirrhotic patients with MHE (*n* = 24) and those without MHE (NHE, *n* = 29). Table 1 lists the demographic and clinical characteristics of the study participants. Exclusion criteria included a current diagnosis of overt HE or other neuropsychiatric disorders, the use of psychotropic medications, the presence of uncontrolled endocrine diseases and metabolic diseases such as thyroid dysfunction, or recent alcohol abuse (less than six months prior to the study). The diagnosis of OHE was based on the West Haven criteria^15^. MHE was diagnosed using the Psychometric Hepatic Encephalopathy Score (PHES), which is comprised of a battery of neuropsychological assessments including the digit symbol test, number connection test A, number connection test B, serial dotting test, and line tracing test. The patient with PHES score ≤ 5 was diagnosed as MHE. Details about the PHES examination and MHE diagnosis have been described previously^20,21^.

**Table 1:** Demographic and clinical features of the study cohort (cirrhotic patients with and without minimal hepatic encephalopathy, MHE and NHE).

Characteristics	NHE patients (*n* = 29)	MHE patients (*n* = 24)	*P*-value
Age (years)	52.6 ± 9.7	50.6 ± 8.9	0.46
Sex (male/female)	24/5	20/4	0.96 (χ^2^-test)
Education (years)	8.3 ± 3.2	8.7 ± 2.7	0.64
Etiology of cirrhosis (HBV/alcoholism/HBV + alcoholism/other)	21/3/2/3	14/5/2/3	—
Child–Pugh stage (A/B/C)	19/8/2	4/14/6	0.001
Previous episode of overt hepatic encephalopathy (no/yes)	19/10	10/14	0.08 (χ^2^-test)
PHES test			
Final PHES (score)	−0.6 ± 2.2	−7.8 ± 3.3	<0.001
Number connection test A (seconds)	39.3 ± 10.8	55.7 ± 17.6	<0.001
Number connection test B (seconds)	74.6 ± 26.9	127.8 ± 63.1	<0.001
Serial dotting test (seconds)	46.7 ± 9.6	64.0 ± 18.2	<0.001
Digit symbol test (raw score)	41.1 ± 12.8	28.2 ± 9.5	<0.001
Line tracing test (raw score)	141.4 ± 34.0	192.5 ± 46.6	<0.001

### MRI acquisition

A 3-T MR scanner (Siemens, Verio, Germany) was used to acquire high-resolution T1-weighted images with a magnetization-prepared rapid gradient echo (MPRAGE) sequence. Image acquisition parameters were as follows: time to repetition (TR) = 1900 ms, time to echo (TE) = 2.48 ms, flip angle = 9 °, field of view (FOV) = 256 mm × 256 mm, matrix = 256 × 256, number of sagittal slices = 176, and slice thickness = 1 mm.

### MRI processing

Image processing was performed using Statistical Parametric Mapping software (SPM8) (http://www.fil.ion.ucl.ac.uk/spm/software/spm8/). In brief, the standard unified segmentation model in SPM8 was used to separate the structural MRI images into gray matter, white matter, and cerebrospinal fluid. Then, the Diffeomorphic Anatomical Registration Through Exponentiated Lie algebra (DARTEL) approach was employed to generate a GM template from all the images^22^ and the template was spatially registered to the tissue probability map in standard Montreal Neurological Institute space. Following this affine registration, each gray matter MR image was non-linearly warped to the above GM template with a 1.5-mm cubic resolution. The GM volume of a single voxel was calculated by multiplying the GM map by the non-linear determinants derived from the spatial normalization step. Finally, the resulting images were refined by smoothing with an 8 mm^3^ full-width at half-maximum (FWHM) kernel.

### Support vector machine analysis

Compared to other classification algorithms, SVMs have good performance and generalization capability when processing small-sample data^23,24^. Through the kernel transformation, SVMs can map the input objects into a higher dimension space. In order to make the classification accuracy as high as possible, a hyperplane needs to be selected to maximize the margin of separation between distinct classes. The key problem for SVMs is how to construct the optimal hyperplane.

**Figure 1:**
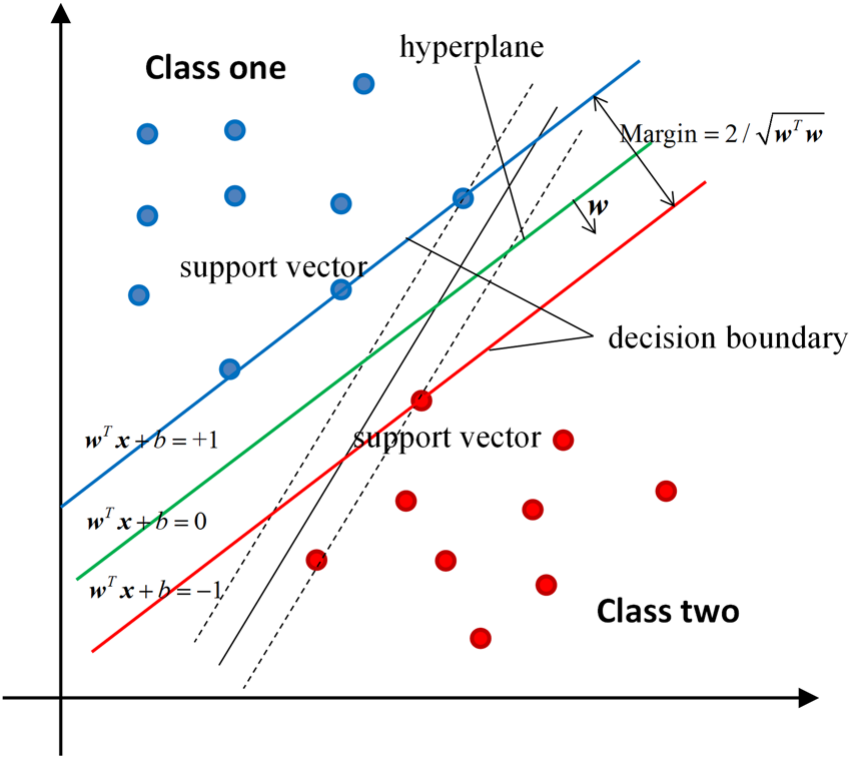
A schematic diagram demonstrating the SVM concept with a linear kernel. The optimal hyperplane is defined by ${{\boldsymbol{w}}}^{T}{\boldsymbol{x}}+b=0$. The sample whose feature ***x*** satisfies the condition ${{\boldsymbol{w}}}^{T}{\boldsymbol{x}}+b\ge +1$ was classified as NHE, while the sample whose feature ***x*** satisfies the condition ${{\boldsymbol{w}}}^{T}{\boldsymbol{x}}+b\le -1$ was classified as MHE.

Assuming a binary classification problem, the input training data has $m$ samples in the form of $< {{\boldsymbol{x}}}_{i},\,{y}_{i} >$, where ${{\boldsymbol{x}}}_{i}$ is an $n$-dimensional vector and $\,{y}_{i}$ is the class label. The optimal hyperplane that separates the given data is then defined as

11 $${y}_{i}=f({{\boldsymbol{x}}}_{i})={{\boldsymbol{w}}}^{T}\Phi ({{\boldsymbol{x}}}_{i})+b,$$

where ***w*** is the “normal vector” perpendicular to the hyperplane, *b* is the offset parameter, Φ is the function of nonlinear transformation, and *T* represents the matrix transpose. Through mathematical derivation, the SVM classifier with the maximum margin can be obtained by optimizing the following function:

1.2 $$\begin{array}{c}Min\frac{1}{2}{{\boldsymbol{w}}}^{T}{\boldsymbol{w}}+C\mathop{\sum }\limits_{i=1}^{m}{\xi }_{i}\\ subject\,to\,{y}_{i}({{\boldsymbol{w}}}^{T}\Phi ({{\boldsymbol{x}}}_{i})+b)\ge 1-{\xi }_{i},i=1,\cdots m,\,{\xi }_{i}\ge 0,\end{array}$$

where ${\xi }_{i}$ is the “slack variable” representing the amount by which each data point deviates from the separation margin, and $C$ is a predetermined constant that controls the balance between the training errors and the misclassification tolerance. Once the “normal vector” ${\boldsymbol{w}}$ and the offset parameter *b* in Eq. (1.2) are calculated, the classification (class label *y*_*i*_) can be predicted for a new sample based on Eq. (1.1). Accordingly, as shownin Fig. 1, when the parameters ***w*** and *b* were calculated, the decision boundary could be described by the equations ***w***^*T*^***x*** + *b* = +1 and ***w***^*T*^***x*** + *b* = −1. These decision boundaries were chosen in order to achieve the maximum margin separating the two classes. The data points lying on the decision boundaries are called “support vectors”.

In this study, the SVM algorithm was carried out using the PRoNTo software (Pattern Recognition for Neuroimaging Toolbox, version 2.1, http://www.mlnl.cs.ucl.ac.uk/pronto/prtsoftware.html)^25^. Each T1-weighted structural image was considered one data point in a high-dimensional space defined by the GM volume (GMV) value. In this high dimensional space, the linear decision boundaries classified the brain scans based on their class label (i.e., the NHE and MHE groups). Specifically, the classifier was trained by providing the samples in the form of $\langle {{\boldsymbol{x}}}_{i},{y}_{i}\rangle$ to find the optimal hyperplane, where ${{\boldsymbol{x}}}_{i}$ represented the input GMV feature and ${y}_{i}$ was the class label (NHE and MHE). The optimal hyperplane was computed based on the varying patterns of GMV values across each T1-weighted image.

We chose a linear kernel over a non-linear kernel for several reasons. Firstly, non-linear kernels do not improve prediction accuracy in the high-dimensional space^26,27^. More importantly, a linear kernel reduces the risk of over-fitting, can greatly increase computational efficiency, and permits whole-brain classification without dimensionality reduction^28^. The similarity matrix was pre-computed using the linear kernel in the PRoNTo software and was then provided to the SVM classifier. The elements in the similarity matrix were calculated as the “dot product” of the input GMV features in the high-dimensional space. Then, the SVM classifier can extract the weight vector (i.e. the “normal vector” ***w***) as an SVM discrimination map. The weight metric (W*i* in Table 2) indicates the strength of the contribution of the GMV feature to the SVM classifier. In our study, we set the parameter *C* = 1 according to previous neuroimaging studies^29,30^. It is noted that the several factors (i.e. individual age, sex, and education level), were included as covariates and regressed out using PRoNTo software, before building the SVM model.

**Table 2:** Brain regions contributing to the identification of MHE vs. NHE.

Cluster size (voxel number)	Gray matter region	Brodmann area	MNI coordinates			W*i* (×10^−3^)
			x	y	z	
**NHE group > MHE group**						
1115	Left Inferior Parietal Lobule	40/7	−30	−49.5	42	9.998
369	Left Middle and Superior Occipital Gyrus	19/18	−27	−81	19.5	5.695
795	Left Middle and Inferior Temporal Gyrus	38/20	−34.5	0	−45	5.679
622	Left Middle and Superior Temporal Gyrus	22/21	−52.5	−40.5	0	5.580
936	Left Inferior Frontal Gyrus	9/6	−55.5	−4.5	22.5	5.508
259	Right Superior and Middle Occipital Gyrus	18/19	24	−85.5	21	5.007
265	Left Middle Frontal Gyrus	6	−24	−4.5	51	5.001
261	Right Fusiform Gyrus	20/36	43.5	−30	−22.5	4.856
331	Right Cerebellum Posterior Lobe		21	−66	−49.5	4.797
246	Left Middle Frontal Gyrus	9	−34.5	31.5	30	4.659
255	Right Inferior and Middle Frontal Gyrus	9/6	39	4.5	33	4.655
349	Right Putamen and Pallidum		21	10.5	−4.5	4.564
259	Right Middle Frontal Gyrus	9	34.5	33	28.5	4.548
616	Left Superior and Middle Frontal Gyrus	11/10	−25.5	48	−15	4.350
306	Right Precuneus and Posterior Cingulate Gyrus	7/31	4.5	−55.5	34.5	4.220
213	Left Supramarginal Gyrus	40	−55.5	−43.5	30	4.095
218	Right Calcarine	30	24	−63	7.5	4.053
316	Right Middle and Superior Frontal Gyrus	10/11	39	54	−3	4.031
208	Left Putamen and Pallidum		−22.5	7.5	−1.5	4.023
296	Right Cerebellum Posterior Lobe		24	−82.5	−34.5	3.755
302	Left Cerebellum Posterior Lobe		−28.5	−79.5	−28.5	3.597
**NHE group < MHE group**						
3159	Left Precentral and Postcentral Gyrus	4/6/3	−25.5	−19.5	70.5	−8.455
1837	Bilateral Thalamus		−7.5	−27	4.5	−7.987
1943	Left Lingual Gyrus	18/17/19	0	−90	−18	−6.352
841	Right Precentral and Postcentral Gyrus	4/5/3	27	−33	70.5	−5.644
376	Left Supramarginal Gyrus and Superior Temporal Gyrus	39/22	−46.5	−54	19.5	−5.563
972	Left Cerebellum Posterior Lobe		−25.5	−43.5	−49.5	−5.342
548	Right Cuneus and Precuneus	31/7	18	−64.5	28.5	−5.026
244	Right Cerebellum Posterior Lobe		24	−42	−49.5	−5.019
827	Right Middle and Inferior Occipital Gyrus	19/18	43.5	−69	−13.5	−4.910
440	Left Middle Occipital Gyrus and Middle Temporal Gyrus	39/19	−42	−76.5	16.5	−4.865
716	Left Insula	13	−37.5	−3	−4.5	−4.776
333	Bilateral Rectus	25	1.5	22.5	−22.5	−4.448
274	Left Postcentral Gyrus	2	−57	−30	31.5	−4.308
725	Left Cerebellum Anterior Lobe		−24	−34.5	−27	−4.127
248	Left Inferior Temporal Gyrus	20	−51	−22.5	−30	−4.048
346	Right Inferior Temporal Gyrus	20	43.5	−10.5	−39	−4.001

Note: The above brain regions were identified by setting the classification threshold to ≥30% of the maximum weight vector scores. The first column lists only clusters larger than 200 voxels. W*i* (reported in the last column) is the weight of each cluster centroid, i.e., the value that indicates the relative contribution of the GMV feature to the SVM-based classification.

The “leave-one-out” cross-validation strategy was adopted in accordance with previous studies^31,32^, which excludes a single subject for testing and uses the remaining subjects for training. Every subject was excluded once to evaluate classification performance. This procedure was applied to all subjects in order to assess the overall accuracy of the SVM^23^. A permutation test (permutations = 1000 times) was applied to determine the statistical significance of the classification accuracy^33,34^.

We analyzed the correlation between the test margin and the PHES results using Pearson correlation analysis. The test margin was computed by projecting the input GMV feature onto the “normal vector” of the hyperplane. Accordingly, a larger absolute value of the test margin meant that the subject lay further away from the hyperplane.

## Results

MHE patients performed significantly worse in all five subtests of the PHES assessment (resulting in a lower final score), indicating significant cognitive deficits compared to the NHE subjects.

**Figure 2:**
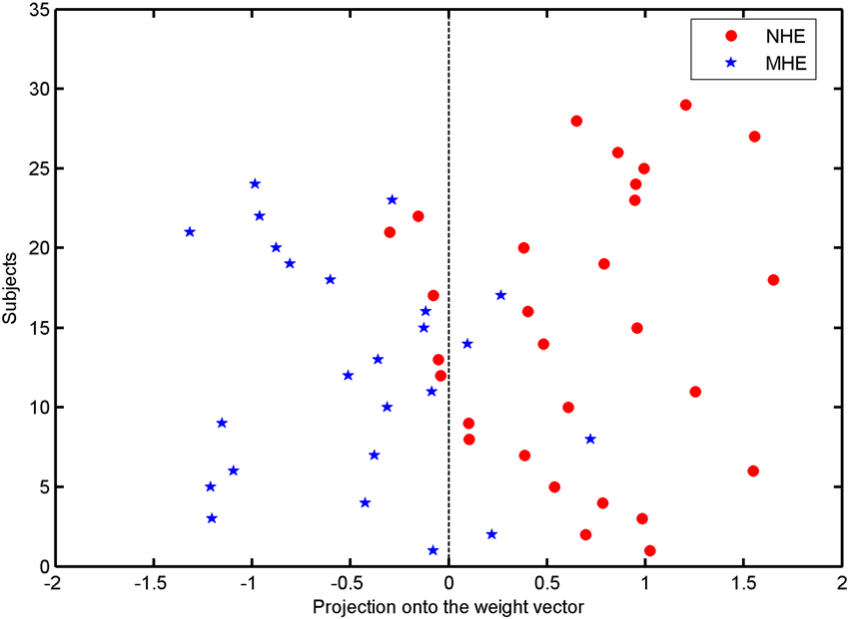
A classification plot comparing NHE patients (n = 29) and MHE patients (n = 24) using the GMV-based discrimination map generated from the T1-weighted MRI scans. The overall accuracy was 83.02% (*P* = 0.001), with a sensitivity of 83.33% and a specificity of 82.76%.

**Figure 3:**
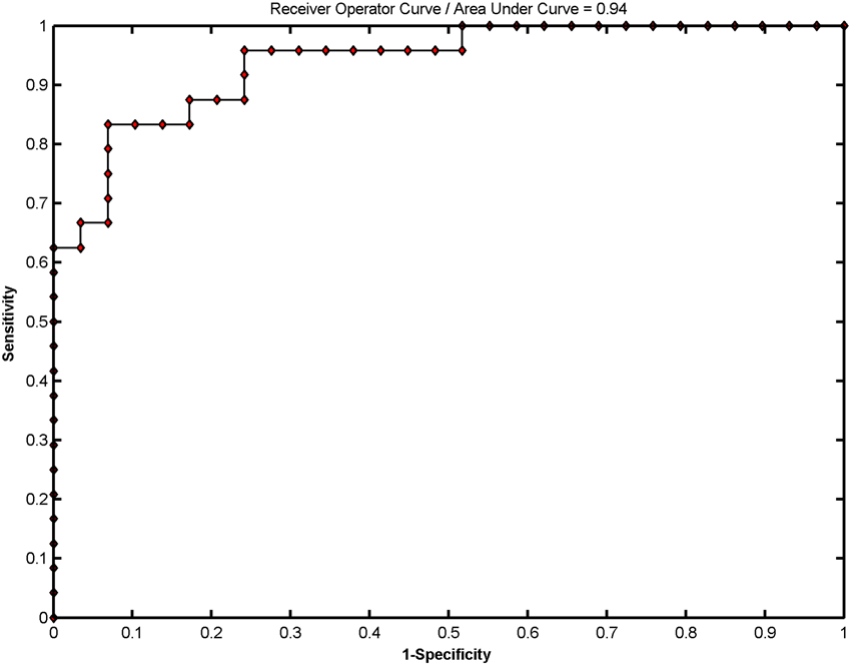
Receiver operating characteristic (ROC) curve showing the classification performance.

Figure 2 shows the SVM classification performance based on GMV between the 29 NHE subjects and the 24 MHE patients. The overall accuracy rate was 83.02% (*P* = 0.001), with a sensitivity of 83.33% and a specificity of 82.76%. As shown in Fig. 3, the area under the receiver operating characteristic (ROC) curve was 0.94, indicating a high possibility of correctly discriminating between the NHE and MHE individuals. Pearson correlation analysis indicated a positive correlation between the test margin and the PHES results (*r* = 0.647, *P* = 1.6 × 10^−7^). Taken together, these results suggested that when the PHES score is far from diagnostic criteria, the subject is unlikely to be misclassified.

**Figure 4:**
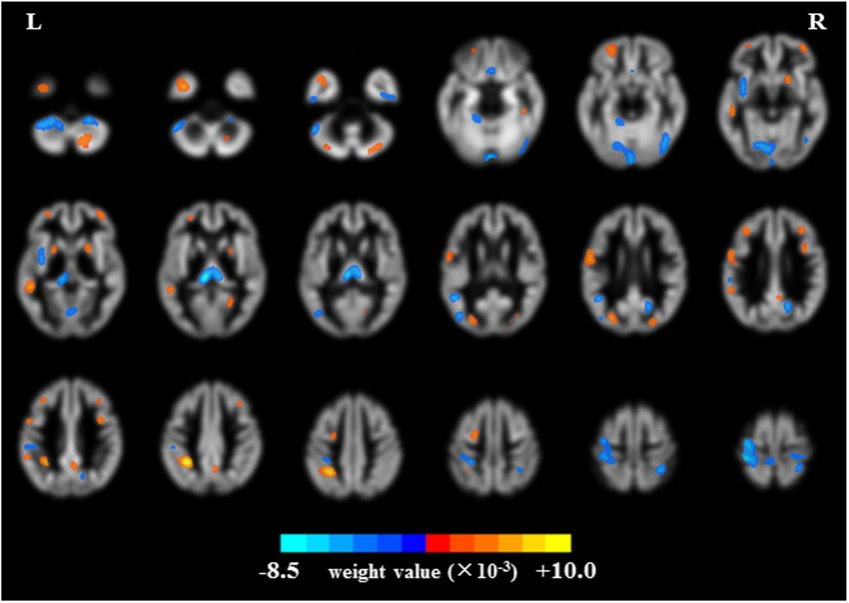
Brain regions classified as MHE and NHE based on gray matter volumetry. The threshold was set to ≥30% of the maximum weight vector scores, and only clusters larger than 200 voxels are shown. The color bar indicates the weight value from the SVM classification, with warm colors (positive weights) representing higher parameter values in NHE subjects and cold colors (negative weights) representing higher parameter values in MHE subjects.

We identified the GM regions that were more associated with MHE or more associated with NHE by setting the threshold to ≥30% of the maximum weight vector scores, as per previous studies^30,35^. Those GM regions with a high absolute value of W*i* had a higher discriminant power between groups. Specifically, GM regions with positive weight values were stronger contributors to recognizing individuals in the NHE group and those with negative weight values were stronger contributors to recognizing individuals in the MHE group (Table 2). Figure 4 uses a color map to indicate the GM regions with positive values (warm colors) and negative values (cold colors) in the discrimination map. The regions that contributed to identifying NHE included the bilateral frontal lobe, bilateral putamen and pallidum, bilateral middle and superior occipital gyrus, bilateral cerebellum posterior lobe, left middle and superior temporal gyrus, left middle and inferior temporal gyrus, left inferior parietal lobule, left supramarginal gyrus, right precuneus and posterior cingulate gyrus, right fusiform gyrus, and the right calcarine. GM regions that identified the MHE group included the bilateral thalamus, bilateral precentral and postcentral gyrus, bilateral inferior temporal gyrus, bilateral rectus, left lingual gyrus, left insula, left cerebellum posterior and anterior lobe, left occipital-temporal junction area, left parietal-temporal junction area, right middle and inferior occipital gyrus, right cuneus and precuneus, and the right cerebellum posterior lobe.

## Discussion

In this study, SVM classification analysis with regional GMV as the indicator yielded 83.02% accuracy (83.33% sensitivity and 82.76% specificity) in classifying the MHE and NHE groups, suggesting the usefulness of gray matter volumetry in identifying early-stage hepatic encephalopathy among cirrhotic patients. Given that GM structural abnormalities exacerbate in stages as HE progresses in cirrhotic patients, and the changes of GM volume and thickness are correlated with cognitive impairments in cirrhosis, it is not unanticipated that gray matter volumetry is successful in differentiating between MHE and NHE diagnoses^6,18,19^. The PHES was designated as the current “gold standard” for MHE diagnosis^15,36^, although its disadvantages are also noted^37^, such as the reliance on the considerable motor activity and the existence of learning effect across the multiple tests. The GM volumetry may be helpful to overcome these disadvantages and play the important role in the assisted diagnosis. In terms of GMV data, the most informative regions were the bilateral frontal lobe, bilateral lentiform nucleus, bilateral thalamus, bilateral sensorimotor areas, bilateral visual regions, bilateral temporal lobe, bilateral cerebellum, left inferior parietal lobe, right precuneus, and the right posterior cingulate gyrus. All of these areas have been frequently reported to be affected by liver dysfunction that often induces energy metabolism disorders and deposition of neurotoxic substances in the brain^38–41^.

The GM regions that contributed to the identification of NHE patients had significantly higher GMV values in control NHE subjects than in the MHE subjects. This reduction of GMV that we observed in the MHE group reflected the previously reported regional atrophy in MHE^6,17,18,42^. For example, cirrhotic patients with MHE have consistently shown a loss of gray matter in both cortical and subcortical structures, such as the frontal and parietal lobes, limbic areas, and striatum^6,42^, and all of these regions were identified in the discrimination map obtained by our SVM procedure. Additionally, this decreased GMV occurred in several brain networks such as the frontoparietal network, the default mode network, and the primary and secondary visual networks. Therefore, network-oriented, regional GM atrophy may also be able to predict the relevant neurological dysfunctions that are common in MHE, such as executive dysfunction, attention deficits, and impaired visuospatial ability^9,43–45^. Similarly, MHE-associated neuronal loss in the basal ganglia and frontal lobe may induce the disintegration of cortico-striatal circuits subserving motor and cognitive processes^46^, and the reduction in cerebellar volume can affect sensorimotor processing in cirrhotic patients with MHE.

The brain regions that contributed to the identification of MHE subjects showed significantly higher GMV in MHE patients compared with NHE subjects. In agreement with this result, previous studies also revealed similar enlargements in these specific GM regions in cirrhotic patients. For example, cirrhotic patients with cognitive impairment have demonstrated a significant increase in cortical thickness in the bilateral lingual and parahippocampal gyrus, right posterior cingulate cortex, precuneus, peri-calcarine sulcus, and the fusiform gyrus^47^. In addition, cirrhosis is often accompanied by an increase in thalamic volume^19,42,48^, so much so that increased GMV in the thalamus has been regarded as an additional characteristic of MHE. Accordingly, it was not unexpected to find that GM regions, such as the bilateral thalamus, bilateral precentral and postcentral gyrus, bilateral inferior temporal gyrus, bilateral occipital lobe, bilateral cerebellum, left insula, and right precuneus, were identified in our study by SVM classification in the discrimination map.

It is important to note that the mechanisms underlying increased GMV in MHE are not well understood. One possible reason may be the diffuse, low-grade, cerebral edema related to Alzheimer’s type II astrocytes during chronic liver disease^47,49^. The existence of both decreases and increases in GMV in MHE may reflect brain structural reorganization due to chronic liver failure. This MHE-associated neural plasticity possibly represents a compensatory mechanism to balance the negative influences of neural metabolic abnormalities.

Despite the compelling results of our study, its limitations are three-fold. 1) The relatively small sample size restricts the statistical power of the results. Accordingly, we encourage future studies to validate the classification potential of GMV using a larger number of study subjects. 2) The MHE patients in our study exhibited mild heterogeneity in terms of the etiology of their cirrhosis and their history of overt HE. This may introduce bias in the classification results since these factors can induce varying degrees of structural and functional impairments in the brain^6,48,50^. 3) While we only examined the discriminative potential of gray matter changes in MHE, mapping abnormal white matter alterations may also be useful to diagnose MHE^51^, which should be investigated in future studies.

In summary, we successfully differentiated between cirrhotic patients with and without MHE using gray matter volumetry and an SVM classification system. The brain regions with the highest discriminant power included both cortical and subcortical structures. Therefore, our findings suggest that regional changes in GMV can be employed as a biomarker to detect MHE in cirrhotic patients.
